# Clinical Experiences with Radiation Induced Thyroid Cancer after Chernobyl

**DOI:** 10.3390/genes2020374

**Published:** 2011-05-31

**Authors:** Christoph Reiners

**Affiliations:** Department of Nuclear Medicine, University of Würzburg, Oberdürrbacherstr.6, D-97082 Würzburg, Germany; E-Mail: reiners_c@klinik.uni-wuerzburg.de; Tel. +49 931 201 55000; Fax: +49 931 201 655001

**Keywords:** Chernobyl, children, thyroid cancer, advanced stages, treatment, prognosis

## Abstract

The risk of developing thyroid cancer increases considerably after exposure to external or internal radiation, especially in children below the age of 10. After the Chernobyl reactor accident, the yearly incidence of childhood thyroid cancer in Belarus increased to approximately 40 per 1.000.000 in girls and to roughly 20 per 1.000.000 in boys compared to approximately 0.5 cases per 1.000.000 prior to the accident. Typically, young children with thyroid cancer after radiation exposure present in ≈95% of the cases as papillary cancers, in ≈50% as invasive tumors growing outside the thyroid capsule, in ≈65% with lymph node metastases and in ≈15% with distant metastases. A joint Belarusian-German project starting in April 1993 that combined treatment with surgery and radioiodine was organized in 237 selected children from Belarus who were exposed to the Chernobyl fallout and had advanced stages of thyroid cancer. The study group included 141 girls and 96 boys. Their median age at the time of the accident was 1.7 years; whereas the median age at the time of diagnosis was 12.4 years. With the exception of two cases with follicular histology, the majority of the patients had been diagnosed with papillary thyroid cancers. In 63%, the tumor had grown outside the thyroid capsule and invaded the tissue of the neck (pT4). Nearly all of the selected cases (96%) showed-up with lymph node metastases (pN1) and 43% of the patients with distant metastases mainly to the lungs (pM1). In 58% of the children, complete remissions of thyroid cancer could be achieved until December 31st 2010 and in 34% of the children, stable partial remissions; in the remaining 8% of the patients, partial remissions were observed. The risk of radiation-induced thyroid cancer increased considerably in children and adolescents who were affected by the Chernobyl reactor accident. In spite of the fact, that thyroid cancers in young children seem to behave more aggressively than in older patients, the results of combined treatment with thyroidectomy, radioiodine therapy and thyroid hormone replacement are excellent.

## 1. Historical Perspective

It is well known from the past, that external radiation can induce thyroid diseases such us hypothyroidism, autoimmunity, nodules and cancer [1]. Several longitudinal cohort studies provide clear evidence of the long-term risks of thyroid cancer and other neoplasms after thyroid exposure to radiation [2]. According to a comprehensive review by Ron *et al.* [3], the excess relative risk for thyroid cancer after medical irradiation per Gy amounts to approximately 4.5. With respect to the exposure to ionising radiation after a nuclear reactor accident, the observations in long-term survivors from the atomic bomb detonations in Japan are appropriate [4,5]. A systematic analysis of age dependence proved that there is a significant increase of thyroid cancer risk in the age group of 0–9 years in atomic bomb survivors [6]. A lesser, but still highly significant risk was found in adolescents from 10–20 years, however, no significant increase of thyroid cancer risk was observed in adults above the age of 20. This is not surprising, since age dependent thyroid dose factors for I-131 range from 36 in newborns to 1.4 in adults according to model calculations [7].

## 2. The Chernobyl Accident

During the night from April 25th to 26th 1986, a very severe reactor accident happened at the nuclear power plant of Chernobyl 30 km south of the border between Ukraine and Belarus. The reactor core exploded and caught fire, and the burning graphite could not be extinguished until May 9th 1986. Due to the long-lasting fire, large amounts of radioactivity were released during 10 days. The radioactivity was transported with the prevailing winds from the northern parts of the Ukraine to Belarus and the western part of Russia and later to Scandinavia and parts of Western Europe. Belarus has been most heavily contaminated with 70% of the released activity. Very high contaminations have been found in people evacuated from the near surroundings of the Chernobyl Power Plant and in the inhabitants of the Gomel area with corresponding thyroid doses by radioiodine isotopes. Iodine blocking of the thyroid was not offered to the population and the children were not prohibited to drink milk contaminated with radioiodine. Mean doses for children below the age of 8 from these locations ranged from 0.6–3.1 Gy and for adults from 0.2–0.7 Gy [8].

## 3. Epidemiological Studies after Chernobyl Regarding Thyroid Cancer

**Figure 1:**
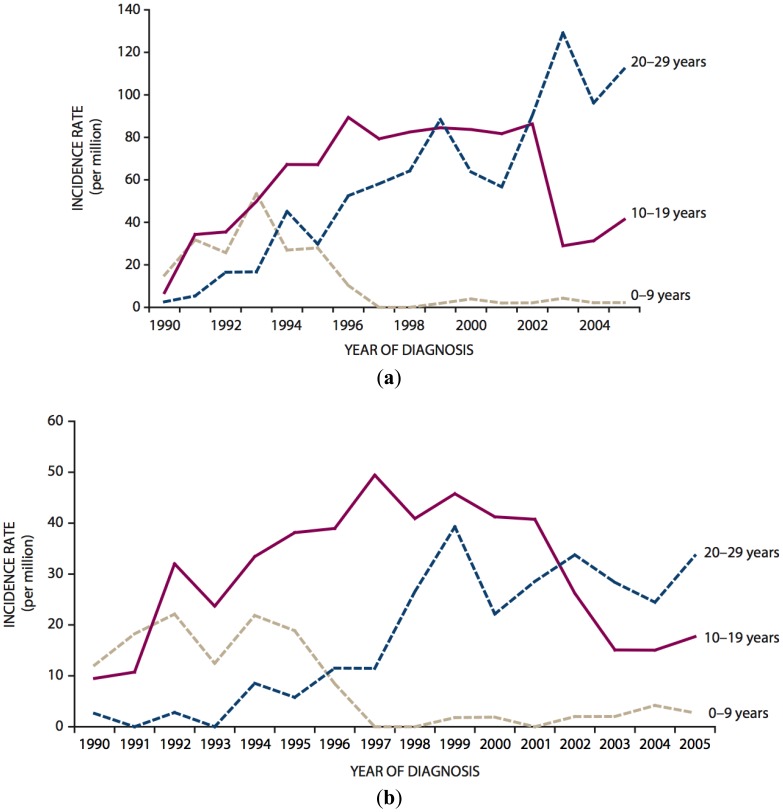
Thyroid cancer incidence rates for different age groups (age at diagnosis) of the total Belarusian population [11]: (a) females; (b) males.

According to a comprehensive review published by UNSCEAR on the occasion of the 25th anniversary of the Chernobyl accident, the main tremendous health effect of radiation from the accident observed until today is a dramatic increase of the incidence of thyroid cancer in persons exposed as children or adolescents [8–11]. This increase was first observed in the early 1990s in Belarus and continues until now in the most contaminated areas of Belarus, Ukraine and the Russian Federation [8–14]. To illustrate this, Figure 1 shows the temporal trends of thyroid cancer in children (0–9 years), adolescents (10–19 years) and young adults (20–29 years) in the general population of Belarus following the accident [11]. By 1993, the incidence of childhood thyroid cancer had increased to approximately 40 per 1.000.000 in girls and to roughly 20 per 1.000.000 in boys compared to approximately 0.5 cases per 1.000.000 per year prior to the accident. As those who were children at the time of the accident have aged (by 2005, even the very youngest had reached adulthood), childhood thyroid cancer rates have declined to near zero and parallel increases in the incidence of thyroid cancer in adolescents and later in young adults have been seen.

According to the recent UNSCEAR Report, 5.127 cases of thyroid cancer were observed between 1991 and 2005 for the whole of Belarus and the most affected regions of the Russian Federation in the age group below the age of 14 in 1986 [11].

At the time of the Chernobyl accident, some experts supposed that radioisotopes of iodine had a lesser carcinogenic potential than external irradiation of the thyroid. However, the excess relative risks derived in the case-control and cohort-studies available up to now show that the risk for thyroid cancer after internal exposure by radioiodine is very similar to the well-known risks after external exposure (see Section 1).

There is some indication that nutritional iodine deficiency at the time of exposure may increase the risk of developing thyroid cancer after incorporation of radioiodine [15,16]. On the other hand, prolonged stable iodine supplementation in the years after exposure may reduce this risk [8]. Experiences from Poland prove that timely preventive thyroid blocking with a high dose of stable iodine (50 mg) to children can very effectively reduce the risk of developing thyroid cancer after exposure to the Chernobyl fallout [17].

It has been claimed that malignant thyroid tumors after external irradiation typically present as papillary cancers in more than 85% of exposed children and adolescents [18]. However, comparison with other data from the literature proves that papillary histology per se is typical for thyroid cancer in childhood and adolescents with and without radiation exposure. Tumor extension and the prevalence of lymph node and distant metastases are associated inversely with age at the time of radiation exposure in children after Chernobyl [18–20]. Typically, children with thyroid cancer after radiation exposure present in ≈95% of the cases as papillary cancers, in ≈50% as invasive tumors growing outside the thyroid capsule, in ≈65% with lymph node metastases and in ≈15% with distant metastases [13,18,21].

## 4. Childhood Thyroid Cancer in Belarus

### 4.1. Ultrasound Screening

Using high resolution ultrasound scanners with 7.5 MHz probes, thyroid nodules with a diameter of 5 mm can be detected. Screening programs in heavily contaminated areas of Belarus revealed prevalences of thyroid cancer in children of 0.6% in 1990 and 0.3% in 1993 [22]. For comparison, the expected prevalence in the western world without radiation exposure is approximately 0.15%. Diagnostic criteria for malignancy in childhood thyroid cancer patients are nodule size, hypoechogenicity and irregularity of the outline, subcapsular location of the lesion and increased peri-intranodular vascularisation [22].

### 4.2. Surgical Treatment

Demidchik *et al.* reported about 740 consecutive patients younger than 15 who underwent surgery and follow-up observations in the Thyroid Cancer Center Minsk, Belarus between August 1985 and October 2003 [13]. Among the 738 patients with non-medullary thyroid cancer, radiation history was documented in 681 (92%) individuals.

All the patients underwent surgery, mostly total thyroidectomy (58%) with simultaneous selective neck dissection (82%), and were maintained on levothyroxine suppressive therapy with a mean dose of 2.0–2.5 μg/kg of body weight. Radioiodine therapy for thyroid remnants, lymph node or lung metastases was performed in 464 (63%) cases. The tumors were classified according to the TNM staging system of UICC, 6th edition [13].

At diagnosis, most patients had solitary, usually asymptomatic tumors measuring less than 2 cm in the largest diameter corresponding to T1 stage (n = 537, 73%) with a very high frequency of lymph node metastases (n = 573, 78%). Distant metastases to the lungs were detected on planar X-rays in 17 (3%) of the patients.

During follow-up, recurrent disease was diagnosed in 204 (28%) of the patients. The main sites of recurrence were the lungs (18%) or lymph nodes (14%) whereas other sites (thyroid remnants, bone, soft tissue or CNS) were uncommon and usually associated with positive neck nodes or pulmonary metastases.

In all of the patients with relapses and other selected cases with advanced disease, radioiodine therapy followed in Essen and later Wuerzburg, Germany (see Chapter 4.3).

### 4.3. Radioiodine Therapy in Germany

A joint Belarusian-German project starting in April 1993 that combined treatment with surgery and radioiodine was launched in a subgroup of patients depicted in Section 4.2. Thyroid surgery was performed in the Center for Thyroid Tumors in Minsk, Belarus [13,18], and radioiodine therapy followed in Germany at the Universities of Essen (until the end of 1994) and afterwards the University of Wuerzburg [18].

The selection criterion for treatment was advanced disease (local invasiveness, lymph node and/or distant metastases). The follow-up period ends in December 2010, now comprising nearly 18 years [23].

#### 4.3.1. Patients

237 children from Belarus with most advanced stages of thyroid cancer were selected for treatment in Germany [23]. The study group included 141 girls and 96 boys. Their median age at the time of the accident was 1.7 years; whereas the median age at the time of diagnosis defined as surgical removal of the primary tumor was 12.4 years. With the exception of two cases with follicular histology, the majority of the patients had been diagnosed with papillary thyroid cancers (235 cases). In 63%, the tumor had grown outside the thyroid capsule and invaded the tissue of the neck (stage pT4). Nearly all of the cases (96%) showed-up with lymph node metastases (stage pN1). In 43% of the patients, distant metastases were detected mainly by I-131 whole-body scanning. The distant metastases were located in the lungs (103 cases) and additionally in the bone (2 cases) and the brain (1 case). Nearly all of the patients with lung metastases presented with disseminated miliary spread (96%), whereas only 4% of the children showed localized focal lesions.

#### 4.3.2. Radioiodine Treatment

In total, 1068 courses of radioiodine treatment in Germany were applied during the treatment period [23]. 136 children without distant metastases received between 1 and 6 treatment courses with radioiodine (median 2). In patients with distant metastases (n = 101) the number of treatment courses ranged from 2 to 12 (median 5). The activity of radioiodine in the patients without distant metastases ranged from 1.0 to 26.1 GBq (median 7 GBq) and in patients with distant metastases, the therapeutic activity ranged from 4.4 to 63.6 GBq (median 21 GBq). The mean interval between 2 consecutive treatment courses was 4.6 months.

#### 4.3.3. Treatment Response

*Complete remissions* were defined as negative whole-body scans and negative serum tumor marker thyroglobulin (not higher than the detection limit of 1 ng/mL). The combination of negative whole-body scans and measurable thyroglobulin levels in the low range up to 10 ng/mL was defined as *stable partial remissions*. In patients with thyroglobulin > 10 ng/mL, therapy response was classified as *partial remission*, if imaging by CT and/or ultrasound showed shrinkage of the tumor, intensity of radioiodine uptake decreased and/or thyroglobulin levels were lowered. In addition, we looked at recurrences and mortality.

The follow-up time between the last course of I-131 therapy and the actual control in Wuerzburg or Minsk ranged from 2.2 to 16.5 years (median 10.1 years).

The results of radioiodine treatment in 236 children with advanced differentiated thyroid cancer from Belarus, who had been treated or followed-up respectively in Germany between the 1st of April 1993 and the 31st of December 2010 can be summarized as follows [23]:

In 58% of the children complete remissions of thyroid cancer could be achieved up to now (negative whole-body scan and negative thyroglobulin). In 34% of the children, we were able to observe stable partial remissions (negative whole-body scans and measurable thyroglobulin < 10 ng/mL). In the remaining 8% of the patients, partial remissions were observed (decrease of tumor volume, of intensity of radioiodine uptake and/or of thyroglobulin serum level).

In patients without distant metastases, complete remissions were observed much more frequently (71%) than for patients with distant metastases (40%). Up to now, no single case of recurrence was detected and only one patient died because of pulmonary fibrosis, presumably induced by radioiodine and pretreatment with the cytostatic drug bleomycin.

## 5. Discussion

Today, the increase of thyroid cancer incidence in children and adolescents from Belarus, Ukraine and Russia affected by the Chernobyl accident is no more a matter of debate, with a projected number of approximately 15.000 cases over 50 years [8,9,11]. Since the prognosis of thyroid cancer is very good (see below), the estimated number of cancer deaths presumably will range between 300 and 1.200 at maximum.

The great majority of post-Chernobyl tumors are papillary cancers. D.Williams [24] pointed out that radiation-induced thyroid cancers are not more aggressive than sporadic cancers and that there is no radiation–specific molecular marker.

The prognosis of thyroid cancer in children and adolescents quoad vitam is excellent. According to the combined data of the US SEER registry, the 30 year survival rate in patients less than 20 years old with papillary thyroid cancer is 92% [25], whereas the follow-up data of the Mayo Clinic as a single center show a considerably better survival of 98% after 40 years [26]. Jarzab *et al.* [27] reviewed the data of 1200 children reported in the literature, revealing survival rates between 97% and 100% after a follow-up of more than 10 years. All the studies prove that age is an important prognostic factor; in spite of the frequently aggressive cancers at presentation in young children, their prognosis is no worse than in older children and adolescents [18,28].

Recurrences, however, occur frequently in childhood thyroid cancer with reported relapse rates between 20% and 40% [13,21,26,27]. Papillary cancers developing after internal exposure to radioiodine do not display specific risk factors for recurrence different from those in sporadic papillary tumors [29].

It is well known, that papillary histology and complete resection of the thyroid and the lymph nodes have a positive prognostic impact with respect to the prevention of relapses [21,24,27,30,31]. In addition, Handkiewicz-Junak [30] proved convincingly, that remnant ablation with radioiodine reduces the number of recurrences significantly by a factor of 3.2. Recently, our group could demonstrate that the success of postoperative I-131 therapy in young Belarusian patients with thyroid cancer after Chernobyl depends on the radiation absorbed dose to the blood and the thyroglobulin level [32].

It has also been shown that treatment results in childhood thyroid cancer do not differ according to whether or not the patient was exposed to radiation [13,21,18,33].

A specific therapeutic challenge in children with thyroid cancer is lung metastases, which frequently present as disseminated “miliary” spread. Our experiences with a large cohort of such patients and data from the literature sho, that it is difficult to achieve complete remissions in a considerable proportion of the cases [34–36]. Since the long-term results in patients even with incomplete remissions are excellent and there is a risk for radiation induced lung fibrosis after repeated courses of radioiodine [36], we underline the statement of Pawelczak *et al.* [35] that “*it is prudent to use caution in the repeated administration of I-131 to such patients to ensure that adverse effects of therapy do not cause more harm than good in a disease that has an overall favourable natural course*” [37].

Up to now, no reliable threshold can be given with respect to the prevention of I-131 therapy-induced pulmonary fibrosis. However, we did not observe it in patients who received less than a cumulative activity of 24 GBq of I-131 because of lung metastases [36]. In patients in stable partial remission, an increase of the specific tumor marker thyroglobulin will indicate progression and the necessity to proceed with further courses of radioiodine therapy.

Contrary to the deterministic effect of radiation induced pulmonary fibrosis, no threshold exists for stochastic long-term side effects of radioiodine therapy because secondary cancers (e.g., leukemia, salivary gland cancer, breast cancer) also have to be taken into account [37,38].

## 6. Conclusions

The risk of radiation-induced thyroid cancer increased considerably in children and adolescents affected by the Chernobyl reactor accident. In spite of the fact that thyroid cancers in young children seem to behave more aggressively than in older patients, the results of combined treatment with thyroidectomy, radioiodine and thyroid hormone replacement are excellent.
